# Supplementary material for: Thermochemical nonequilibrium flow analysis in low enthalpy shock-tunnel facility
Source: PLoS One. 2020 Oct 7;15(10):e0240300. doi: 10.1371/journal.pone.0240300 (PMC7540898; doi:10.1371/journal.pone.0240300)
Supplement: Attachment — Submitted filename: Response_to_reviewers.pdf [file pone.0240300.s001.pdf]

Reply to the Reviewer's Comments

Manuscript ID: PONE-D-20-06935

Type: Research Article

Title: Thermochemical nonequilibrium flow analysis in low enthalpy shock-tunnel facility

Authors: Sanghoon Lee, Ikhyun Kim, Gisu Park (Korea Advanced Institute of Science and Technology), Jong Kook Lee (Agency for Defense Development), and Jae Gang Kim (Sejong University; Corresponding author)

We would like to express our sincere appreciation for the reviewers' valuable comments and suggestions on the manuscript. We have addressed all the comments and suggestions raised. We hope that the revised manuscript is now acceptable by the reviewers.

Reviewer #1

Comment 1:

The flow which is treated as quasi-one-dimensional flow is right or not?

Response:

The three-dimensional flow calculations have an advantage of the performance analysis of the shock-tunnel facility[1]. However, the computational costs of the three-dimensional calculations are enormous to analyze the thermochemical nonequilibrium phenomena of the shock-tunnel flows. In the work by Nomelis et al.[2], the two-dimensional axi-symmetric calculations of the shock-tunnel flow were preformed, and it was compared with the results by the quasi-one-dimensional calculations and the measured data of the shock-tunnel facility. The comparisons confirmed that the quasi-one-dimensional calculations are acceptable to analyze the thermochemical nonequilibrium of the shock-tunnel flows. Hence, the thermochemical nonequilibrium analysis of the shock-tunnel flows has been widely performed by using the quasi-one-dimensional calculations[3].

In the revised manuscript, the explanation of the quasi-one-dimensional calculations are added in **'Introduction'** on the lines 28-41 in page 3.

[1] Luo K, Wang Q, Li J, Li J, Zhao W. Numerical nodeling of a high-enthalpy shock tunnel driven by gaseous detonation. *Aerosp Sci Technol*. 2020;104. doi:10.1016/j.ast.2020.105958.
[2] Nompelis I. Computational study of hypersonic double-cone experiments for code validation. Department of Aerospace Engineering and Mechanics, University of Minnesota, Minnesota, USA: Ph.D. Dissertation; 2004.
[3] Jacobs PA. Quasi-one-dimensional modeling of a free-piston shock tunnel. *AIAA J*. 1994;32(1):137{145. doi:10.2514/3.11961.

facility without affecting the flow. Numerical simulations are therefore commonly performed to understand and diagnose the thermochemical nonequilibrium state inside the shock-tunnel facility and the hypersonic free-stream conditions at the nozzle exit. In numerical simulations, the three-dimensional flow calculations have an advantage of the performance analysis of the shock-tunnel facility [6]. However, the computational costs of the three-dimensional calculations are enormous to analyze the thermochemical nonequilibrium phenomena of the shock-tunnel flows. In the work by Nomelis et al. [7], the two-dimensional axi-symmetric calculations of the shock-tunnel flow were preformed, and it was compared with the results by the quasi-one-dimensional calculations and the measured data of the shock-tunnel facility. The comparisons confirmed that the quasi-one-dimensional calculations are acceptable to analyze the thermochemical nonequilibrium of the shock-tunnel flows. Hence, the thermochemical nonequilibrium analysis of the shock-tunnel flows has been widely performed by using the quasi-one-dimensional calculations. [8] In the present work, the Q1D calculations are employed to analyze the thermochemical nonequilibrium of the shock-tunnel flows for the numerical efficiency.

Comment 2:

The three-temperature model is proposed by author or referenced other authors?

Response:

The three-temperature model is proposed by authors. There have been several attempts to model the thermochemical nonequilibrium phenomena using a multi-temperature model. In the present three-temperature model, unlike the previous thermochemical nonequilibrium models, the electron-electronic-N_2 vibrational temperature and species vibrational temperature of O_2 and NO are treated as the separated nonequilibrium modes.

Comment 3:

Treating the vibrational nonequilibrium of O_2 and NO separately is not suitable

Response:

As a reviewer commented, in the previous two-temperature model, the vibrational nonequilibrium modes of O_2 and NO do not be treated as separately. The objective of the previous two-temperature model is to describe the nonequilibrium phenomena at the high-enthalpy flows above 8 MJ/kg. In the high enthalpy environments, the dissociation of O_2 occurs rapidly, and the influence of the species vibrational nonequilibrium of O_2 and NO is not significant. However, in low-enthalpy shock tunnel flows below 8 MJ/kg, where the stagnation temperature is less than 6,000 K, the dissociation rate of O_2 is mild, and it couples with the species vibrational nonequilibrium of O_2. Therefore, a more sophisticated consideration of species vibrational nonequilibrium is required.

In the low-enthalpy shock tunnel flows, the species vibrational relaxation time of O_2 differs from that of N_2, and the species vibrational nonequilibrium of NO is not significant because the amount of NO produced from the exchange reaction is relatively small. Hence, in the present work, the species vibrational nonequilibrium of O_2 and NO are treated as one species vibrational nonequilibrium mode, and treated separately from the electron-electronic-N_2 vibrational nonequilibrium mode.

Comment 4:

The conclusions should include more information, data is better.

Response:

As the reviewer suggested. We add more information which can be inferred from the present work in **'Discussion and conclusions'** on the lines 565-581 in pages 25-26.

In the thermochemical nonequilibrium analysis in low enthalpy shock-tunnel flows, discernible differences were observed in the nonequilibrium temperatures behind the incident and reflected shock waves. The electron-electronic-vibrational temperature of the 3-T model relaxes more slowly to the equilibrium temperature than the 2-T model behind the incident and reflected shock waves. Depending on the flow conditions, the relaxation time for electron-electronic-vibrational temperature of the 3-T model is 1.25 to 3 times slower than that of the 2-T model. This is because, in the 3-T model, the electron-electronic-vibrational temperature is mainly influenced by the vibrational relaxation of N_2, and the vibrational relaxation time of N_2 is much slower than that of O_2 and NO in low enthalpy flows. In the chemical reactions behind the incident and reflected shock waves, O_2 dissociation is dominant, and the reaction speed predicted by the 3-T and 2-T models is different. The differences of nonequilibrium characteristics of N_2 and O_2 are also obviously apparent in the nozzle expanding flows where the vibrational temperature freezes. In 3-T model, depending on the flow condition, the frozen electron-electronic-vibrational temperature at the nozzle exit is 15–75% higher than that in the 2-T model. On the other hand, the species vibrational temperature of the 3-T model is around 10–40 % lower than the electron-electronic-vibrational temperature in the 2-T model. Due to the rapid freezing of electron-electronic-vibrational temperature in the 3-T model, some part of energy is locked as nonequilibrium energies so that the trans-rotational temperature and velocity in the 3-T model are lower than those of 1-T and 2-T models.

Reviewer #2

Overall comments:

This is a good paper for this journal and should be accepted with some revisions. A very detailed analysis, but in real shock tube flows, the three-dimensional effects are going to swamp the thermochemical issues associated with the two-temperature model.

Response:

As a reviewer commented, there are three-dimensional effects in real shock-tube which significantly affect the flow, especially in terms of performance and measurement aspects. In the present work, we limited our scope to quasi-one-dimensional flow for the detailed analysis of shock-tunnel flows in thermochemical nonequilibrium aspects.

In the revised manuscript, the more explanation of the quasi-one-dimensional calculations are added in **'Introduction'** on the lines 28-41 in page 3.

facility without affecting the flow. Numerical simulations are therefore commonly performed to understand and diagnose the thermochemical nonequilibrium state inside the shock-tunnel facility and the hypersonic free-stream conditions at the nozzle exit. In numerical simulations, the three-dimensional flow calculations have an advantage of the performance analysis of the shock-tunnel facility [6]. However, the computational costs of the three-dimensional calculations are enormous to analyze the thermochemical nonequilibrium phenomena of the shock-tunnel flows. In the work by Nomelis et al. [7], the two-dimensional axi-symmetric calculations of the shock-tunnel flow were preformed, and it was compared with the results by the quasi-one-dimensional calculations and the measured data of the shock-tunnel facility. The comparisons confirmed that the quasi-one-dimensional calculations are acceptable to analyze the thermochemical nonequilibrium of the shock-tunnel flows. Hence, the thermochemical nonequilibrium analysis of the shock-tunnel flows has been widely performed by using the quasi-one-dimensional calculations. [8] In the present work, the Q1D calculations are employed to analyze the thermochemical nonequilibrium of the shock-tunnel flows for the numerical efficiency.

Comment 1 (Abstract):

‘The proposed three-temperature model was able to describe the different thermochemical nonequilibrium characteristics of N_2 and O_2 behind the incident and reflected shock waves and at the nozzle exit’. I’m not sure what is meant by different. Please clarify specific fluid mechanic of thermochemical phenomena.

Response:

As the reviewer suggested, we added the physical explanation of the present results in ‘**Abstract**’.

shock-tunnel facility. The results of the thermochemical nonequilibrium analysis of the low enthalpy shock-tunnel flows suggest that the nonequilibrium characteristics of N_2 and O_2 need to be treated separately. The vibrational relaxation of O_2 is much faster than that of N_2 in low enthalpy condition, and the dissociation rate of O_2 is manly influenced by the species vibrational temperature of O_2. The proposed three-temperature model is able to describe the thermochemical nonequilibrium characteristics of N_2 and O_2 behind the incident and reflected shock waves, and the rapid vibrational freezing of N_2 in nozzle expanding flows.

Comment 2 (Introduction):

‘Unfortunately, it is difficult to measure all flow properties, because the flow duration is typically only a few hundreds of milliseconds. You mean s few milliseconds. A few hundred would be terrific.

Response:

It is our mistake to express as “a few hundreds of milliseconds”. As the reviewer suggested, we corrected the sentence on the lines 24-25 in page 3.

Unfortunately, it is difficult to measure all flow properties, because the flow duration is typically only a few milliseconds. It is also difficult to install instruments inside the

Comment 3 (Thermochemical nonequilibrium model):

Darcy-Weisbach friction factor f. This assumes laminar flow, correct? This may not be the case.

Response:

We believe that Darcy-Weisbach friction factor f can be used in the shock-tube flow where the Reynolds number is up to 10^5. Though the turbulent flow cannot be directly considered in quasi-one-dimensional calculation, the viscous effects can be considered. This can be achieved by calculating the wall shear stress in the viscous source term using Darcy-Weisbach friction factor f, which can be explicitly determined from Reynolds number. The correlation of Reynolds number and Darcy-Weisbach friction factor f has been extensively investigated in the Reynolds number range of 3000 to 10^8. This method has been generally adopted for quasi-one-dimensional calculation of shock-tunnel flows, for example, L1D code in the university of Queensland. We reflected this comment in the revised manuscript including the equation on the lines 258-263 in pages 13-14.

the determination of shear stress τ_0, the Darcy-Weisbach friction factor f [8, 33, 34] for the pipe flow has been widely used for Q1D calculations [6, 8], and can be represented as follows:

$$\tau_0 = \frac{-\rho f u|u|}{8}. \tag{27}$$

In this study, the Darcy-Weisbach friction factor f for turbulent regime is obtained from Ref [37] for high Reynolds number up to 10^5 as follows:

$$f = \frac{1}{\Lambda}\left[1.8 \log_{10} Re - 1.5147\right]^{-2}, \tag{28}$$

Comment 4 (Thermochemical nonequilibrium analysis in the K1 shock-tunnel facility):

'Na was employed to measure the electron-electronic-vibrational temperature T_{eev}. The electronic temperature was modeled on the emissions spectra of Na, and the concentration of Na was varied between 10 and 100 ppm by volume'. Seeding sodium in the shock tunnel is going to change the reaction rates. Why is this a valid comparison?

Response:

We agree with the reviewer's comment. In line-reversal methods, the metallic seeding species are used to approximately measure the vibrational temperature of diatomic molecules. However, it is found that the concentration of seeding species could enhance the vibrational relaxation process. In the Park's work, which this study benchmarked, the vibrational relaxation time between Na and N_2 was set to physically-fastest time to reflect this matter, and the calculation for the nozzle flow was performed under varying concentration of Na up to 100 ppm. The present study followed this approach, and it can be seen in Fig 3 (b) that the N_2 vibrational temperature changes with the concentration of Na. For conciseness, we have only mentioned about the method used in Park's work, but have not included the results of the Park's work. We reflected this matter by modifying the Fig 3 (b) which now includes the calculation results from Park's work, for the code-to-code validation as well as the comparison with experimental measurements on the lines 302-313 in page 16.

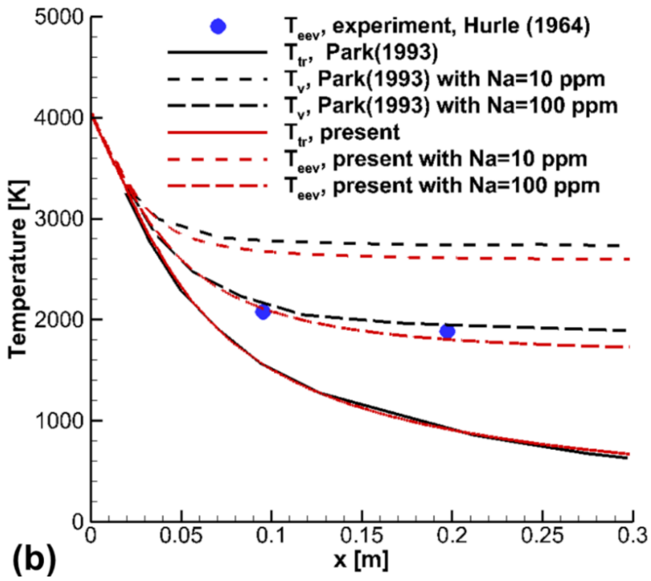

(b)

Fig 3(b) shows a comparison between the calculated electron-electronic-vibrational temperature T_{eev} in the nozzle expanding flows and the experimental data [50] and the results calculated by Park [31]. In experiments performed by Hurle et al. [50], the spectrum-line reversal method with Na was employed to measure the electron-electronic-vibrational temperature T_{eev}. The electronic temperature of Na was modeled based on the emission spectra of Na. The measured electronic temperature was then treated as the same as the vibrational temperature of N_2, because the interchange of N_2 vibrational energy and electronic energy of a metallic particle is rapid and highly effective, so that electronic temperature of Na easily equilibrates with the N_2 vibrational temperature [30]. As seed species can enhance the rate of vibrational relaxation process [51], Park [31] employed the physically fastest vibrational relaxation time for the collision between N_2 and Na and calculated the nozzle flow. In that calculation, the concentration of Na was varied between 10 and 100 ppm by volume. In the present work, the nozzle flow calculations were conducted using the initial conditions of p_0=5 atm and T_0=4,500 K. The nozzle shape and the vibrational relaxation time for Na collisions were obtained from the work by Park [31]. A mesh of

Comment 5 (Thermochemical nonequilibrium analysis in the K1 shock-tunnel facility):

'Reservoir and nozzle exit conditions of K1 shock-tunnel flows'. Why are only some conditions completely listed. Please list all conditions.

Response:

As a reviewer suggested, we corrected the **Table 5** in page 25.

Table 5. Reservoir and nozzle exit conditions of K1 shock-tunnel flows.

	Case A		Case B		Case C		Case D
	Exp. [19]	Calc.	Exp. [20]	Calc.	Exp. [17]	Calc.	Calc.
Shock-tube part							
p_2 (kPa)	52.7±1.9	51.3	14.2±0.8	16.7	616.7±18.3	601.5	29.8
p_5 (kPa)	276±3.0	279	110±6.9	114	3344±89.8	3391	221
T_5 (K)	-	4011	3750±215	3938	-	1686	3893
h_5 (MJ/kg)	-	4.23	-	6.13	-	1.87	7.32
u_{shock} (km/s)	1.96±0.5	2.01	2.50±0.3	2.52	1.15±0.02	1.19	2.44
Nozzle part							
h_e (MJ/kg)	-	4.61	-	7.56	1.68±0.04	1.64	7.21
M_∞ $(-)$	-	7.90	-	6.06	6.06±0.75	5.87	5.822
p_{pitot} (kPa)	-	5.62	-	2.50	85.0±1.69	80.7	2.648
T_{tr} (K)	-	209	-	462	-	225	507
T_{eev} (K)	-	4006	-	2940	-	1610	2949
T_v (K)	-	619	-	966	-	1238	1033
