# Supplementary material for: Thermochemical nonequilibrium flow analysis in low enthalpy shock-tunnel facility
Source: PLoS One. 2020 Oct 7;15(10):e0240300. doi: 10.1371/journal.pone.0240300 (PMC7540898; doi:10.1371/journal.pone.0240300)
Supplement: Attachment — Submitted filename: Response_to_reviewers(2nd).pdf [file pone.0240300.s002.pdf]

# Reply to the Reviewer's Comments

***Manuscript ID:*** PONE-D-20-06935

***Type:*** Research Article

***Title:*** Thermochemical nonequilibrium flow analysis in low enthalpy shock-tunnel facility

***Authors:*** Sanghoon Lee, Ikhyun Kim, Gisu Park (Korea Advanced Institute of Science and Technology), Jong Kook Lee (Agency for Defense Development), and Jae Gang Kim (Sejong University; Corresponding author)

We would like to express our sincere appreciation for the reviewers' valuable comments and suggestions on the manuscript. We have addressed all the comments and suggestions raised. We hope that the revised manuscript is now acceptable by the reviewers.

# Reviewer #1

## Comment 1:

*The authors have send the revised version of their article. The quality of the article has improved but some of the remarks haven't been addressed in a satisfactory level.*

*The introduction is still superficial. The reviewer suggests the author should refer some works about non-equilibrium condensation model, such as:*

*[International Journal of Multiphase Flow, 2019(114)180-191.],*

*[Energy Conversion & Management, 2019, 181:159-177.],*

*[doi:10.1016/j.applthermaleng.2019.114388.], and so on.*

*Besides, the reason of non-equilibrium flow should be explained, the reference maybe could help you*

*[Applied Thermal Engineering. 2020;171:115090.],*

*[doi:10.1016/j.energy.2020.118690.],*

*[doi:10.1016/j.ijmultiphaseflow.2019.103083.].*

## Response:

We appreciate the reviewer's comment for broadening our knowledge. As the reviewer suggested, we modified on lines in **'Introduction'** on the lines 23-31 in pages 2-3.

> as shock stand-off distance differ [4,5]. In addition to this, rigorous understanding for the flowfield is also important in practical aspects. For example, for the humid air could condense after the rapid expansion through the nozzle [6,7]. In nozzle flow where the velocity gradient is large, the difference in time between the inertial restoration and temperature restoration is significant, which leads to thermodynamically nonequilibrium condensation [8,9]. It has been reported that the liquid mass fraction, which would influence the measurement accuracy [10], is mainly determined by nonequilibrium condensation [11,12]. Therefore, the accurate prediction of flowfield is important in analyzing the test results.

6. Pouring AA. Thermal choking and condensation in nozzles. Phys Fluids. 1965;8(10):1802–1810. doi:10.1063/1.1761112.

7. Pouring AA. Study of the unsteady condensation of moist air in shock tube. J Therm Sci. 2004;13(3):235–244. doi:10.1007/s11630-004-0037-0.

8. Zhang G, Zhang X, Wang D, Jin Z, Qin X. Performance evaluation and operation optimization of the steam ejector based on modified model. Appl Therm Eng. 2019;163. doi:10.1016/j.applthermaleng.2019.114388.

9. Zhang G, Dykas T, Yang S, Zhang X, Li H, Wang J. Optimization of the primary nozzle based on a modified condensation model in a steam ejector. Appl Therm Eng. 2020;171. doi:10.1016/j.applthermaleng.2020.115090.

10. Herring GC. Mach-number measurement with laser and pressure probes in humid supersonic flow. AIAA J. 2008;46(8):2107–2108. doi:10.2514/1.36107.

11. Zhang G, Zhang X, Wang F, Wang D, Jin Z, Zhou Z. Design and optimization of novel dehumidification strategies based on modified nucleation model in three-dimensional cascade. Energy. 2019;187. doi:10.1016/j.energy.2019.115982.

12. Zhang G, Zhang X, Wang F, Wang D, Jin Z, Zhou Z. Numerical investigation of novel dehumidification strategies in nuclear plant steam turbine based on the modified nucleation model. Int J Muliph Flow. 2019;120. doi:10.1016/j.ijmultiphaseflow.2019.103083.
